# Characteristics and Prognosis of “Acute Promyelocytic Leukemia-like” Nucleophosmin-1-Mutated Acute Myeloid Leukemia in a Retrospective Patient Cohort

**DOI:** 10.3390/biomedicines12102282

**Published:** 2024-10-09

**Authors:** Vasiliki Papadopoulou, Giulia Schiavini, Gregoire Stalder, Valentin Basset, Jacqueline Schoumans, Mitja Nabergoj, Muriel Schaller

**Affiliations:** 1 Hematology Service and Laboratory, Department of Oncology, Lausanne University Hospital, 1011 Lausanne, Switzerland; 2 Hematology Service, Department of Oncology, Cantonal Hospital of Valais, 1951 Sion, Switzerland; 3 Oncogenetics Unit, Hematology Service and Laboratory, Department of Oncology, Lausanne University Hospital, 1011 Lausanne, Switzerland

**Keywords:** AML with *NPM1* mutation, acute promyelocytic leukemia, *IDH1/2* mutations, *TET2* mutations, DNMT3A mutations, *FLT3* mutations, Ras-pathway mutations

## Abstract

**Background:** AML with *NPM1* mutation is the largest subcategory of AML, representing about 35% of AML cases. It is characterized by CD34 negativity, which suggests a relatively differentiated state of the bulk of leukemic blasts. Notably, a significant subset of NPM1-mutated AML cases also exhibit HLA-DR negativity, classifying them as “double-negative”, and mimicking, therefore, the CD34^−^ HLA-DR^−^ immunophenotype of acute promyelocytic leukemia (APL). **Objectives:** This study focuses on the “acute promyelocytic leukemia-like” (“APL-like”) subset of NPM1-mutated AML, which can be challenging to distinguish from APL at presentation, prior to confirming RARa translocations. We aim to investigate the hematologic and immunophenotypic parameters that may aid to its distinction from APL. Additionally, we explore differences in genetic profile and prognosis between “APL-like” and “non-APL-like” NPM1-mutated AML cases. **Methods:** We conducted a retrospective evaluation of 77 NPM1-mutated AML cases and 28 APL cases. **Results:** Morphological characteristics, hematologic parameters (such as DD/WBC and PT/WBC), and specific immunophenotypic markers (including SSC, CD64, and CD4) can assist in the early distinction of “APL-like” NPM1-mutated AML from APL. Regarding differences in genetic profiles and outcomes between “APL-like” and non-“APL-like” NPM1-mutated AML cases, we observed a significantly higher incidence of IDH1/2 /TET2 mutations, along with a significantly lower incidence of DNMT3A mutations in the “APL-like” subset compared to the non-“APL-like” subset. The frequency of Ras-pathway and FLT3 mutations did not differ between these last two groups, nor did their prognoses. **Conclusions:** Our findings contribute to a comprehensive characterization of NPM1-mutated AML, enhancing diagnostic accuracy and aiding in the detailed classification of the disease. This information may potentially guide targeted therapies or differentiation-based treatment strategies.

## 1. Introduction

Acute myeloid leukemia (AML) with mutation of *NPM1* (NPM1m AML) is a distinct entity, accounting for around 30% of AML cases, with peak incidence in middle age [1,2]. It usually is a “de novo AML”, but may, in some cases, arise as the clonal evolution of a myelodysplastic syndrome (MDS), with *NPM1* mutations being reported in up to 14% of MDS-derived AML [3]. Nucleophosmin-1 (NPM1) is a nucleolar protein believed to participate in the regulation of gene expression via mechanisms such as histone chaperoning and DNA-repair. Additionally, it may be involved in ribosome biogenesis, centrosome duplication, and other intracellular procedures [4,5]. When mutated (in 98% of cases, mutation lies in exon 12 in the C-terminal domain, and in 2% of cases, it lies in exon 5), NPM1 loses its nucleolar localization signal, translocates to the cytoplasm, and either becomes unable to perform its functions or acquires novel ones. Some of its novel functions might correspond to direct binding to specific chromatin targets, which could sustain the active transcription of target genes by inhibiting histone deacetylases. This process may lead to the upregulation of well-known leukemia-driving genes such as *HOXA/B* cluster genes and *MEIS1* [6,7]. However, the exact mechanisms by which *NPM1* mutation provokes disease, and their interaction with coexisting lesions, remain ill-defined. *NPM1* wild-type haploinsufficiency itself appears to provoke myeloproliferative disease in mice (though not AML as a single lesion) [8,9] and to synergize with the overexpression of *MEIS1* [10].

Although the pathogenesis of NPM1m AML is unclear, its typically coexisting mutations are well identified and include *FLT3* alterations, mutations in genes coding for Ras-pathway components, and mutations in the epigenetic factors *TET2*, *IDH1/2*, and *DNMT3A*, with the mechanisms of synergy not being well understood [11]. Current knowledge on the clonal architecture of NPM1m AML shows that *NPM1* mutation is often sub-clonal to *DNMT3A*, *IDH1/2*, or *TET2* mutations, which can pre-exist as clonal hematopoiesis of indeterminate potential [12,13].

Phenotypically, the CD34-negative blast phenotype in NPM1m AML is of particular interest. Only very rare cases express^,^ CD34 and these are usually *FLT3-ITD (internal tandem duplication)*-positive. CD34 expression is also slightly more frequent at relapse [4]. The pathophysiology behind this CD34 negativity is not known. Interestingly, one work has shown that only a minor (undetectable on routine immunophenotyping) CD34^+^ fraction can reproduce the leukemic phenotype in mice [14], while the CD34^−^ fraction cannot. However, there are also data showing the opposite [15]. Clinically detectable *NPM1*-mutated cells probably originate from an immature CD34^+^ *NPM1*-mutated hematopoietic cell, but the leukemic bulk population is detected at a later differentiation stage, in which *HOX* expression is still high, whereas CD34 expression is already silenced. In this context, there is, therefore, only a minor “stem-like” population, which contains leukemia-initiating cells that may expand at relapse, expressing both CD34 and high HOX levels [16,17].

The vast majority of NPM1m AML cases are therefore CD34-negative, and they can be immunophenotypically distinguished in three categories: 1. monoblastic/monocytic (at least 40% of cases): defined by CD64 and HLA-DR positivity and monoblastic/monocytic features on cytology; 2. “acute promyelocytic leukemia-like” (“APL-like”) (approximately 30% of cases): these cases are not only CD34-negative but also HLA-DR-negative, just like APL (“double-negative”); and 3. the remaining 30% of cases: these are CD34-negative but express HLA-DR and do not express CD64 or other classical markers of monocytic differentiation [2,18,19].

This work focuses on “APL-like” NPM1m AML, also known in the literature as “double-negative” (DN) NPM1m AML (CD34^−^ HLA-DR^−^), whose immunophenotype indeed resembles differentiated granulocytic precursors, making it difficult to distinguish from APL, which is, by definition, CD34^−^ HLA-DR^−^. Through retrospective analysis of our cohort of NPM1m AML patients, we aim to address diagnostically and therapeutically relevant questions: Is it possible to distinguish “APL-like” NPM1m AML from APL, based on morphology, hematologic/coagulation, or immunophenotypic parameters? Can it be differentiated immunophenotypically from APL using antigen surface markers? What are the concurrent gene mutations in “APL-like” NPM1m AML, and do they differ from those in “non-APL-like” NPM1m AML? Is prognosis different between these two subsets? Characterizing this “subtype” of NPM1m AML could possibly be useful for tailoring new treatment strategies based on promoting differentiation. Hereafter, “non-APL-like” NPM1m AML will refer to all NPM1m AML cases that express HLA-DR (these include cases with monocytic markers such as primarily CD64, as well as cases with no monocytic markers).

## 2. Materials and Methods

### 2.1. Patients

We identified 77 patients with newly diagnosed NPM1m AML, diagnosed, and/or followed for at least part of their treatment, at the University Hospital of Lausanne, from 2015 until the end of July 2023. Indeed, 75 patients carried the classical NPM1 W288Cfs*12 mutation. The other 2 patients had the translocations t(5;6)(q35;q23) and t(3;5)(q25;q35), respectively, which are known to result, similarly to point mutations, in the cytoplasmic localization of NPM1. At least in the case of t(5;6)(q35;q23), these translocations also display a CD34 negativity of blasts, just like NPM1m AML [20,21]. We also identified 28 APL cases. Table 1 shows the age and *FLT3-ITD/TKD (TKD: tyrosine kinase domain mutation)* positivity ratios of the groups “APL-like” NPM1m AML and “non-APL-like” NPM1m AML at diagnosis, as well as the proportion of cases that underwent allografting and the numbers of cases treated with intensive chemotherapy or other regimens (chemo: intensive chemotherapy, HMA: hypomethylating agent, VEN: venetoclax, FLT3i: FLT3 inhibitor). Table 2 presents the hematologic characteristics of “APL-like” NPM1m AML and of APL cases at diagnosis, which are to be compared, to establish their utility for differential diagnosis between these two entities.

### 2.2. Flow Cytometry

Flow cytometry at diagnosis was performed using combinations of fluorochrome-conjugated antibodies against CD38, CD34, HLA-DR, CD117, CD13, CD33, CD123, CD4, CD56, CD64, CD14, CD300e, CD61, CD15, CD65, CD19, CD10, and CD7 and against cytoplasmic antigens MPO, CD3, TdT, and CD79a after cell permeabilization. All cases of the cohort met the WHO criteria for myeloid lineage of the blasts (which also comprises monoblastic/monocytic cases). Fluorescence values of the different surface/cytoplasmic markers were compared to those of isotype controls, and the expression of the respective markers was measured on the log scale (with scores 1–3). Only side scatter (SSC) measurements were performed on a linear scale (Figure S1).

### 2.3. Oncogenetics Analyses

All cases were analyzed at diagnosis by conventional karyotyping or array-CGH or both; RT-MLPA for fusion transcripts with previously published methods [22]; PCR with FRET probes and melting curve analysis for *NPM1* mutations; and an NGS panel containing 35–41 genes (*ASXL1, BCOR, BRAF, CALR, CBL, CEBPA, CSF3R, DNMT3A, ETV6, EZH2, FLT3, GATA2, HRAS, IDH1, IDH2, JAK2, KIT, KRAS, MPL, NPM1, NRAS, PHF6, PRPF8, PTPN11, RUNX1, SETBP1, SF3B1, SH2B3, SRSF2, STAG2, TET2, TP53, U2AF1, WT1, ZRSR2;* in 01.2023, the panel was expanded to include *BCORL1, CUX1, DDX41, ETNK1, PPM1D, NF1*). (Oncomine™ Myeloid Research Assay, ThermoFisher Scientific (Waltham, MA, USA) + Panel Custom; Ion Torrent, S5XL; Seqpilot Module SeqNext version 5.4.0, Alamut Visual Plus, IARC; Depth > 100×: 98%; Median depth: 3708; Reference genome: GRCh37 Nomenclature: HGVS). The variant allele frequencies (VAFs) of mutated genes at diagnosis were measured with an error margin of 1% on NGS. Figure S2 shows the genes tested and the mutations found in each individual case.

### 2.4. Statistical Analysis

Wilcoxon’s rank-sum test was used to compare the hematologic parameters white blood cell count (WBC), D-dimers (DDs), fibrinogen, prothrombin time (PT), the derived parameters DDs/WBC and PT/WBC, as well as the immunophenotypic parameters (SSC value on linear scale, CD117, MPO, CD38, CD4, CD64 expressions on log scale) and age between “APL-like” NPM1m AML and APL cases. Chi-square tests with Yates’ continuity correction were employed to compare the incidence of *IDH1/2/TET2* mutations, Ras-pathway mutations (*NRAS, KRAS, HRAS, CBL, PTPN11, NF1* [23]), *DNMT3A* mutations, *FLT3-ITD/TKD* mutations, and the frequencies of the treatment regimens received between “APL-like” and “non-APL-like” NPM1m AML cases. Event-free survival (EFS) was defined as the time (from diagnosis) to death or relapse or treatment failure (the latter being defined as failure to achieve CR/CRh/CRi after one cycle of intensive chemotherapy or after two cycles of hypomethylating agent (HMA)+/− venetoclax or after at least one cycle of HMA+/− venetoclax if treatment regime was changed thereafter). Patients evaluable for response but not achieving either CR, CRh, or CRi by the defined milestones and patients who died before the defined milestones without response assessments were considered events at day 1. Patients who were alive but non-evaluable for response during their follow-up period were censored at day 1, as proposed by general guidelines on AML [24]. EFS and overall survival (OS, time from diagnosis to death) were compared between the “APL-like” and “non-APL-like” NPM1m AML groups using the log-rank test. Statistical analyses were performed using the R software (version 4.2.2 (31 October 2022)).

## 3. Results

**Figure 1:**
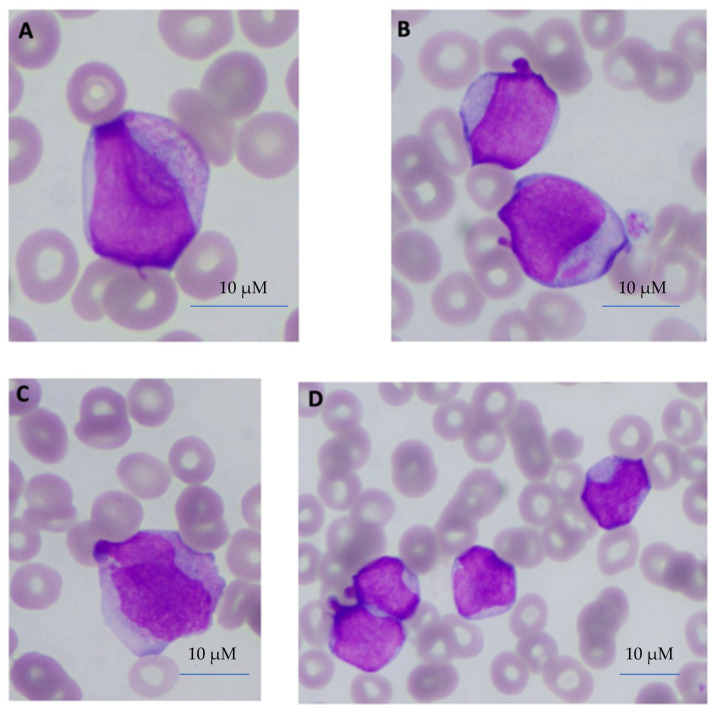
**Morphology of peripheral blood (PB) blasts in APL and in “APL-like” NPM1m AML.** (**A**) Peripheral blast of patient with APL, featuring a typical bilobed nucleus with typically overlapping lobes and an inconspicuous Auer rod, as is often the case in APL in PB. (**B**) Peripheral blasts in “APL-like” (CD34- HLADR-) NPM1-mutated AML case, displaying a prominent Auer rod and a tendency to “cup-like” nuclei. (**C**) Peripheral blast from another patient with APL, showing a typical bilobed nucleus with typically overlapping lobes and non-readily visible Auer rods. (**D**) Peripheral blasts in an “APL-like” (CD34- HLADR-) NPM1-mutated AML case, characterized by typical “cup-like” nuclei.

Twenty-six (26) out of seventy-seven (77) NPM1m AML cases were identified as “APL-like” NPM1m AML immunophenotypically (Figure S1). We aimed to describe potential features that may distinguish “APL-like” NPM1m AML from APL prior to obtaining the results of PCR or FISH for *RARa* translocations. We did not use digital methods for the quantification of features of cellular morphology; however, our observations indicate that, in our cohorts, it is rather not the presence of Auer rods that distinguishes the two entities on peripheral blood morphology. In APL, Auer rods, while present, are very often inconspicuous in the peripheral leukemic promyelocytes and are more readily visible in bone marrow promyelocytes. In contrast, Auer rods in peripheral blasts of “APL-like” NPM1m AML were quite frequent and often more easily observed than in the peripheral promyelocytes of APL (Figure 1A,B). The nuclear morphology of APL, on the other hand, is highly distinctive, characterized by nuclei that are finely folded upon careful inspection in a manner almost unique to the disease. In “APL-like” NPM1m AML, most peripheral blood blasts displayed “cup-like” nuclei (Figure 1C,D).

**Figure 2:**
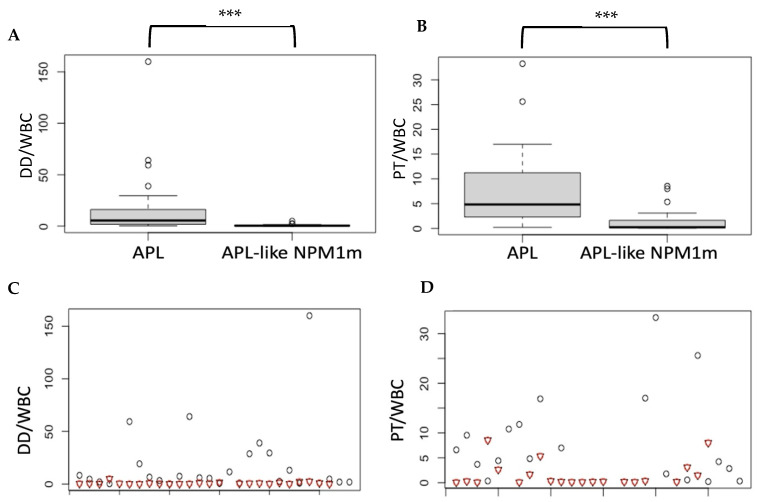
**Distribution and differences in DDs/WBC and PT/WBC ratios between APL and “APL-like” NPM1m AML** (***: *p* < 0.001). (**A**) Box plot of ratios DDs/WBC (mg/L / G/L) in our acute promyelocytic leukemia (APL) cases and “APL-like” NPM1-mutated cases (APL-like NPM1m). Difference is statistically significant between the two groups (*p* < 0.001, Wilcoxon’s rank-sum test). (**B**) Box plot of ratios PT/WBC (s/G/L) in our acute promyelocytic leukemia (APL) cases and “APL-like” NPM1-mutated cases. Difference is statistically significant between the two groups (*p* < 0.001, Wilcoxon’s rank-sum test). (**C**) DDs/WBC ratios of APL cases shown with black circles, and DDs/WBC ratios of “APL-like” NPM1-mutated cases are shown with red triangles. Cases with ratio > 4.92 (mg/L/G/L) are always APL. (**D**) PT/WBC ratios of APL cases are shown with black circles, and PT/WBC ratios of “APL-like” NPM1-mutated cases are shown with red triangles. Cases with ratio > 8.54 (s/G/L) are always APL.

To investigate if the two entities APL and “APL-like” NPM1m AML can be distinguished based on hematologic parameters, before obtaining the result of *RARa* translocations, we collected values for total leukocyte count (WBC), fibrinogen, D-dimers (DDs), and prothrombin time (PT) at diagnosis. It is well known that APL is often oligo-leukocytic, while NPM1m AML can frequently present with hyperleukocytosis; it is, additionally, common knowledge that DIC (disseminated intravascular anticoagulation)/hyperfibrinolysis is a hallmark of APL. Therefore, differences in WBC, fibrinogen, and PT values may be expected and were indeed found to be statistically significant between our two cohorts (28 APL patients and 26 “APL-like” NPM1m AML patients), with median values, respectively, of 2.25 vs. 53.25 G/L for WBC, 1.5 vs. 4.5 g/L for fibrinogen, and 11.7 vs. 13.1 s for PT (none of the patients was anticoagulated on presentation). The difference in DD values between the two cohorts was non-significant (Table 2). The total leucocyte count may depend on the disease’s burden at diagnosis, so if we are to compare coagulation parameters relevant to DIC, it would probably be more accurate to normalize them to WBC rather than compare them as absolute values. We therefore compared DDs/WBC and PT/WBC ratios between the two entities and found statistically significant differences, with much higher median values in APL than in “APL-like” NPM1m AML for both ratios (Table 2). The distribution curves of these two ratios for the two entities are shown in Figure 2A–D. In our cohorts, a DDs/WBC ratio > 4.92 (mg/L/G/L) and a PT/WBC ratio > 8.54 (s/G/L), which correspond to the highest values measured in “APL-like” NPM1m AML cases, were only seen in APL and could thus indicatively serve in favor of an APL diagnosis.

**Figure 3:**
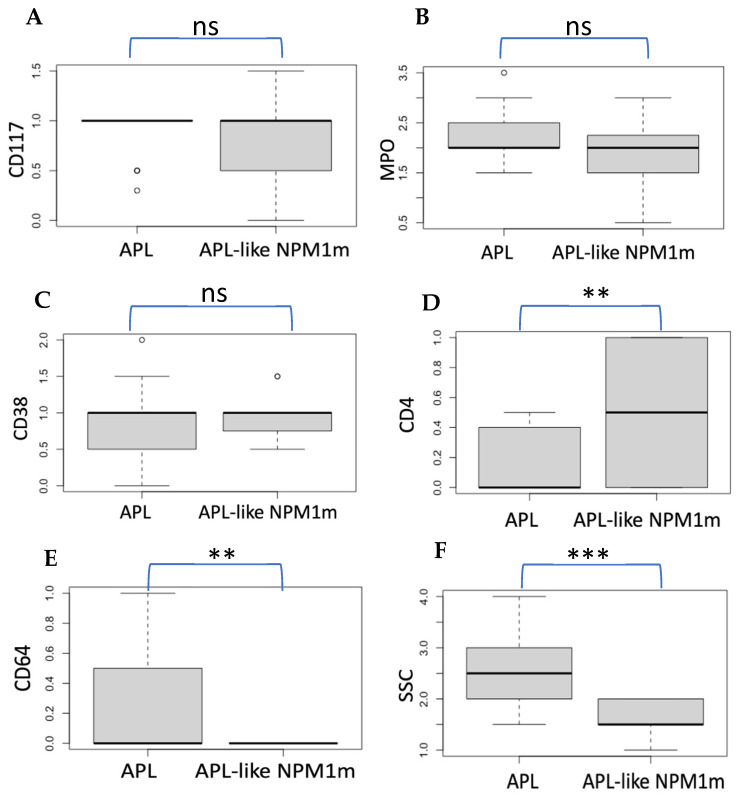
**Differences in expression of immunophenotypic markers between APL and “APL-like” NPM1m-AML.** (**A**–**C**) The expression levels of CD117, MPO, and CD38 did not differ significantly between acute promyelocytic leukemia (APL) cases and “acute promyelocytic leukemia-like” NPM1-mutated AML cases (ns: non-significant, **: *p* < 0.01, ***: *p* < 0.001). (**D**–**F**) Significant differences were observed in the expression of CD4 and CD64 and in SSC values between the two cohorts, with APL cases showing higher CD64 expression and SSC values, and “APL-like” NPM1m AML cases showing higher CD4 expression. Comparisons were made using Wilcoxon’s rank-sum test. It is important to note that “APL-like” NPM1-mutated AML, as immunophenotypically defined in this study (CD34- HLADR-), is “by definition” CD64-negative (CD64-positive NPM1-mutated cases exhibit a monocytic phenotype expressing HLA-DR).

Before addressing the immunophenotypic distinction between APL and “APL-like” NPM1m AML, it is important to note that all of our 77 NPM1m AML cases were indeed CD34-negative as expected, except for 1 case (with a classical *NPM1* type A point mutation), which showed moderate expression of CD34 at diagnosis and relapse. We examined the side scatter (SSC) measurements (reflecting granular complexity of the cytoplasm) and the expression of antigens CD117, MPO, CD38, CD4, and CD64 (Figure 3A–F). There was no significant difference in the expressions of CD117 and MPO, nor in the expression of the early myeloid differentiation marker CD38 between APL and “APL-like” NPM1m AML; both entities likely have their leukemic clones at some stage of differentiation. However, the expression of the markers CD4 and CD64 differed between the two entities: some APL cases expressed CD64 (not expressed in “APL-like” NPM1m AML, as this could categorize the case as monocytic/monoblastic NPM1m AML), while the “APL-like” NPM1m AML group showed a tendency for higher CD4 expression compared to APL. We then examined whether the APL cases expressing CD64 had their population located towards the “monocyte gate” in the SSC/CD45 plot, but this was not the case in the majority of these APL cases. Finally, side scatter (SSC) values differed significantly between APL and “APL-like” NPM1m AML, with APL showing higher median SSC values. This difference reflects greater cytoplasmic granularity in APL, even though in some cases this granularity can be submicroscopic.

**Figure 4:**
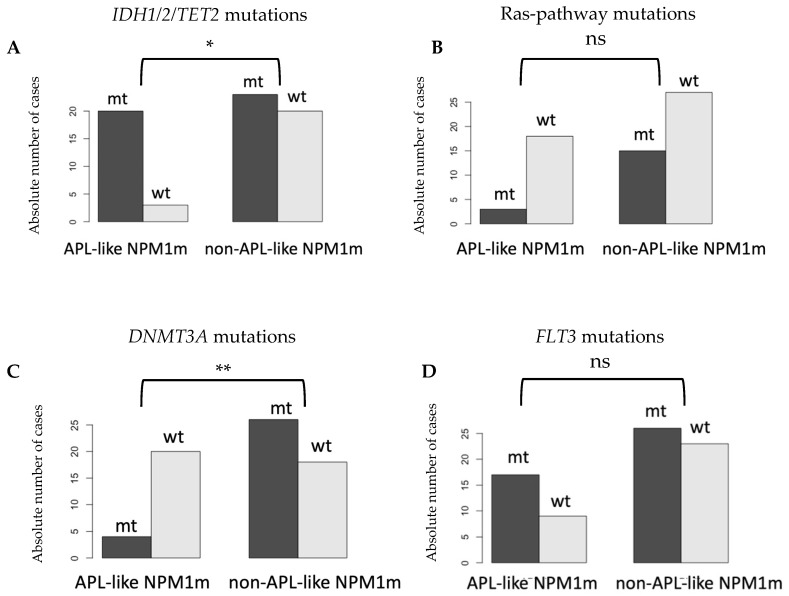
**Differences in the proportion of mutated versus wild-type cases for genes *IDH1/2/TET2*, “Ras-pathway genes”, *DNMT3A,* and *FLT3* among “APL-like” and “non-APL-like” NPM1m AML.** ns: non-significant, *: *p* < 0.05, **: *p* < 0.01. (**A**) Proportion of *IDH1/2/TET2*-mutated cases among “APL-like” NPM1m AML and “non-APL-like” NPM1m AML. “APL-like” NPM1m AML exhibits a significantly higher rate of *IDH1/2/TET2* mutations (chi-square, *p* = 0.0143). (**B**) Proportion of Ras-pathway-mutated cases among “APL-like” NPM1m AML and “non-APL-like” NPM1m AML. No significant difference in the frequency of Ras-pathway mutations (chi-square, *p* = 0.1391) was observed between the two entities. (**C**) Proportion of DNMT3A-mutated cases among “APL-like” NPM1m AML and “non-APL-like” NPM1m AML. “APL-like” NPM1m-AML shows a significantly lower rate of *DNMT3A* mutations (chi-square, *p* = 0.0018). (**D**) Proportion of FLT3-mutated cases among “APL-like” NPM1m AML and “non-APL-like” NPM1m AML. No significant difference in the frequency of *FLT3* mutations (chi-square, *p* = 0.4344) was found between the two entities.

In our attempt to characterize “APL-like” NPM1m AML, we also investigated potential differences between the genetic profiles of “APL-like” and “non-APL-like” NPM1m AML. This question has been previously explored, with partially conflicting results (which will be discussed later). The most frequent concurrent mutations in NPM1m AML occur in *FLT3*, in genes coding for Ras-pathway components, and in the *IDH1/IDH2/TET2* and *DNMT3A* genes; we therefore compared the frequency of these four mutational categories between our “APL-like” and “non-APL-like” NPM1m AML cohorts. There is a rationale for grouping together *IDH1/2* and *TET2* mutations into a single mutational category, different than the one for *DNMT3A* (another gene coding for a component of the epigenetic machinery). The IDH (isocitrate dehydrogenase) enzymes convert isocitrate to a-ketoglutarate (a-KG), within and outside of, the Krebs cycle [25]; a-KG can then serve as a substrate (OH-donor) for dioxygenase-type enzymes, such as histone demethylases and DNA-5-methylcytosine hydroxylases like TET2. As hydroxylation of the 5-methylcytosines of DNA is the first step towards DNA demethylation [26,27], the activity of IDH1/2 enzymes ultimately promotes DNA and histone demethylation by providing a-KG to dioxygenases [28]. Therefore, IDH1/2 and TET2 enzymes possibly serve the same epigenetic pathways at different steps. In contrast, DNMT3A (DNA methyltransferase) activity competes with TETs at methylated somatic enhancers, leading to different effects on the gene expression sets of haemopoietic progenitor cells [29,30]. In our cohorts, we found a significantly higher frequency of *IDH1/2/TET2* mutations in “APL-like” NPM1m AML (almost all cases bearing one such mutant) and a significantly higher frequency of *DNMT3A* mutations in “non-APL-like” NPM1m AML. There was no difference in the frequency of mutations in “Ras-pathway genes” or *FLT3* (including TKD and ITD) between the two groups (Figure 4).

**Figure 5:**
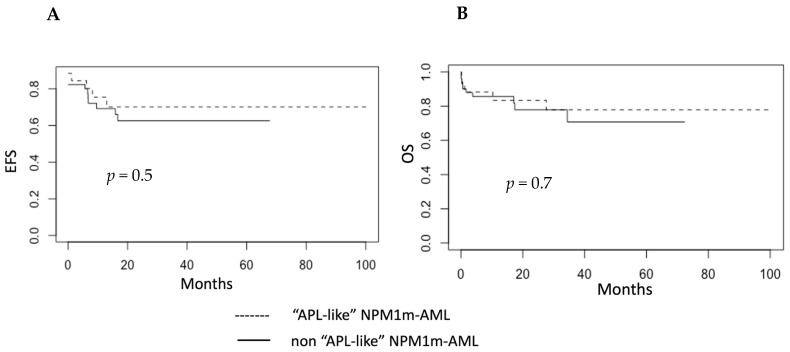
**Event-free survival (EFS) and overall survival (OS) of “APL-like” and “non-APL-like” NPM1m AML in our cohorts.** (**A**) Event-free survival: no statistically significant difference was observed between “APL-like” and “non-APL-like” NPM1-mutated AML cases (*p* = 0.5 log-rank test). (**B**) Overall survival: no statistically significant difference was observed between “APL-like” and “non-APL-like” NPM1-mutated AML cases (*p* = 0.7, log-rank test).

Furthermore, a comparison of the prognosis between our cohorts of “APL-like” and “non-APL-like” NPM1m AML revealed no differences in event-free survival (EFS) or overall survival (OS) (Figure 5A,B). It is important to note that the two cohorts did not differ significantly in terms of the proportion of *FLT3*-mutated cases, patient age distribution, proportion of cases that underwent allografting, or the treatments administered (Table 1).

## 4. Discussion

“APL-like” or “double-negative” (DN) (CD34^−^ HLA-DR^−^) NPM1m AML can be challenging to distinguish from acute promyelocytic leukemia (APL) before obtaining results of FISH or PCR for *RARa*-fusions, as both entities share a similar immunophenotype and may present with Auer rods in peripheral blood leukemic cells. Historically, the coagulation profile of APL is characteristic, often showing prevalent DIC or hyperfibrinolysis, while NPM1m AML is not typically associated with these traits, although they can occur. We aimed to identify laboratory (hematologic and immunophenotypic) parameters that can be used to distinguish “APL-like” NPM1m AML from APL and deemed it reasonable to normalize the relevant parameters to white blood cell counts, as the latter may reflect the total disease burden at diagnosis. Indeed, the parameters DDs/WBC and PT/WBC exhibited statistically significant differences between the two entities. The cutoffs we identified, above which a CD34^−^ HLA-DR^−^ (double-negative) constellation suggests an unlikely diagnosis of “APL-like” NPM1m AML (favoring APL) are presented in the Results section. There have been small case series describing “APL-like” NPM1m AML, confirming that these cases can indeed often present with overt DIC, similarly to APL [31,32]. Additionally, published work indicates that “double-negative” (CD34^−^ HLA-DR^−^) AMLs (not necessarily NPM1-mutated) tend to exhibit a higher propensity to DIC compared to “non-double-negative” AMLs [33]. This last report raises the question if there are other AML types, aside from AML with *NPM1* mutation, that can be “double-negative”; the article itself reveals that over two-thirds of cases of “double-negative” AML were NPM1-mutated and that over 95% of cases had normal karyotype, but does not specify the exact diagnoses in the DN-AML cases which allegedly did not carry *NPM1* mutation. During our study, an Italian group posed similar questions regarding the hematologic/coagulation parameters of immunophenotypically “APL-like” NPM1m AML, comparing them, in a relevant meeting abstract, to those of the remaining NPM1m AML (whereas we compared them to APL). The group found that “APL-like” NPM1m AML showed a significantly greater tendency toward DIC than the immunophenotypically “non-APL-like” NPM1m AML subset, using parameters such as D-dimers, fibrinogen, and vascular events [34].

Immunophenotypically, distinguishing between APL and “APL-like” NPM1m-AML (both of which are double-negative for CD34 and HLA-DR) is challenging, as the expressions of MPO and CD117 are not distinctive. The side scatter (SSC) parameter, which reflects the granular complexity of the cytoplasm, was, however, higher in APL in our cohorts. Additionally, we observed a tendency for more frequent/increased CD4 expression in “APL-like” NPM1m AML compared to APL, although not all our “APL-like” NPM1m AML cases expressed it. This higher CD4 expression in “APL-like” NPM1m AML is supported by previously published data [31]. CD64 was expressed in several APL cases in our cohort; therefore, a CD34-negative HLA-DR-negative AML, that expresses CD64 over log 1 or higher, should be regarded as highly suspicious for APL. In contrast, according to our definition, “APL-like” (“double-negative”) NPM1m AML does not express CD64, as CD64-positive cases fall into the category of monoblastic/monocytic NPM1m AML, which typically expresses HLA-DR. In the literature, CD64 expression in APL is frequently reported, with intermediate mean fluorescence intensities (MFIs), whereas the relatively lower range of MFIs observed in our cohort may be attributed to set thresholds [35,36].

The correlation of phenotype with genotype in NPM1m AML is an area of extensive research, yielding rather conflicting results from data gathered from cohort studies, mouse models, and lately single-cell technologies. The genetic profile of “APL-like” NPM1m AML in our cohort shows that, indeed, it has a much higher frequency of *IDH1/2* or *TET2* mutations compared to the rest of the NPM1m AML cases [18,37,38,39]. Additionally, we confirm that *DNMT3A* mutations are significantly more common in the “non-APL-like” NPM1m AML subset [37,38]. *FLT3* mutations have been shown to correlate with the very rare expression of CD34 in NPM1m AML [17,38]; indeed, in our entire NPM1m AML cohort, the only CD34^+^ case carried *FLT3-ITD*. Ras-pathway gene mutations in NPM1m AML correlated with HLA-DR expression, indicating a “non-APL-like” phenotype, in one published study, and with a monocytic rather than a “double-negative” phenotype in another work, both based on bulk NGS [37,38]. Conversely, in a study conducted on NPM1-mutated knock-in mice, Ras-pathway mutations correlated with a granulocytic bias, while *FLT3* mutations correlated with a monocytic bias [40]. In a single-cell NGS-based analysis, *NRAS* mutations were associated with a non-monocytic phenotype [13]. In our cohort, there was no difference in the frequency of Ras-pathway mutations between the double-negative (“APL-like”) and non-double-negative (“non-APL-like”) subsets of NPM1m AML.

The prognosis of “APL-like” and “non-APL-like” NPM1m AML did not differ in our cohort. It is important to note that this is not a randomized prospective trial, and our patient numbers are relatively low. One study consistently demonstrated higher relapse-free survival in the “double-negative” subset [18,37], while another group reported no difference in a relevant abstract [34]; randomized comparative data are lacking. In discussing prognosis based on immunophenotype in NPM1m AML, it is worth mentioning that the very rare CD34 expression has been associated with worse outcomes [16]. Prognostic comparisons are likely to be more reliable when based on co-mutation profiles rather than immunophenotype, and the former are likely often reflected in the latter. In discussing prognosis based on genetics, the unfavorable prognosis of *FLT3-ITD* in NPM1m AML is, for instance, well established in the formal risk stratifications of AML. High-quality data have shown that it is rather the combination of *DNMT3A* mutation status and *FLT3-ITD* status that define prognosis; with wild-type *DNMT3A*, the presence of *NRAS* or *FLT3-ITD* mutations does not significantly affect the prognosis of NPM1m AML, whereas *DNMT3A*-mutated NPM1m AML shows distinctly unfavorable prognosis in the presence of *FLT3-ITD* (comparing to *FLT3* wild-type cases) and favorable prognosis in the presence of *NRAS* mutations (comparing to *NRAS* wild-type cases) [11,41]. However, we did not compare prognosis among co-mutation profile subgroups of NPM1m AML in the current study.

In summary, our work emphasizes that NPM1m AML is characteristically CD34-negative and focuses on the “APL-like” immunophenotypic subtype of NPM1m AML. We demonstrate that, while awaiting the results of *RARa*-fusions testing, the entities “APL-like” NPM1m AML and acute promyelocytic leukemia (APL) can be distinguished based on specific hematologic indices (DDs/WBC ratio and PT/WBC ratio for non-anticoagulated patients), as well as the expression of CD4 and CD64. Moreover, we find that “APL-like NPM1m AML” carries *IDH1/2/TET2* mutations significantly more frequently, and *DNMT3A* mutations significantly less frequently, than the other NPM1m AML cases. Notably, the prognosis of “APL-like” NPM1m AML was not significantly different from that of the rest of the NPM1m-AML cohort in our study. The limitations of our work include its retrospective nature and the relatively low patient numbers. Additionally, it is important to note that our analysis is based on bulk NGS and not on single-cell analysis and, secondly, that NPM1m AML cases may harbor two or more *NPM1*-mutated subclones with different mutational profiles (and correspondingly possibly different immunophenotypic profiles), which may complicate categorization. In this work, we defined “APL-like” NPM1m AML cases as having a single immunophenotypic population, exhibiting the “double-negative” (CD34- HLADR-) phenotype; cases with two or more immunophenotypic populations including (only one such case was present), or not, a “double-negative” population, were classified in the category of “non-APL-like” NPM1m AML, as our intention was to include purely “APL-like” NPM1m AML in the so-named cohort.

The CD34-negativity of NPM1m AML suggests that this AML type may be preferentially amenable to differentiation therapies. Indeed, menin inhibitors represent an effective differentiation therapy in NPM1m AML, with a well-defined mechanism of action that involves inhibition of the KMT2A–menin interaction [42]. Early data indicated a potential sensitivity of NPM1m AML to all-trans retinoic acid (ATRA) [43]. However, a recent phase III trial failed to show a benefit of ATRA in a comprehensive population of NPM1m AML patients [44]. Nevertheless, the question remains whether the subset of NPM1m AML with a granulocytic bias (“APL-like” NPM1m AML) could specifically benefit to a greater extent from such therapies. Furthermore, it would be interesting to investigate whether IDH1/2 inhibitors (which can also induce a differentiation syndrome [45,46,47]) are preferentially active or more likely to cause differentiation syndrome in (*IDH1/2*-mutated) “APL-like” NPM1m AML rather than in NPM1-wild-type AML with *IDH1/2* mutations.

## Figures and Tables

**Table 1:** Patient numbers, ages, proportion of *FLT3-ITD/TKD*-mutated cases, proportion of cases allografted and treatment regimens among double-negative CD34^−^HLADR^−^ (“APL-like”) and non-double-negative (“non-APL-like”) NPM1-mutated AML cases. *p*-values are provided for comparisons between the two groups regarding age, proportion of FLT3-mutated cases, proportion of allografted cases (Wilcoxon’s rank-sum tests), as well as differences in treatment regimens (chi-square test).

	“APL-like” NPM1m (26)	“non-APL-like” NPM1m (51)	
**Age (median, range)**	61 (28–87)	61 (35–86)	*p* = 0.67
**FLT3mut**	17/26	26/49 (2 not tested)	*p* = 0.43
**Allografted**	13/26	22/51	*p* = 0.74
**Treatment**			*p* = 0.67
**Chemo**	23	38	
**HMA+VEN**	1	3	
**HMA**	0	3	
**HMA+FLT3i**	0	1	
**Unknown**	0	1	
**None**	2	5	

**Table 2:** Hematologic and hemostatic parameters of acute promyelocytic leukemia (APL) patients and of “APL-like” NPM1m AML patients. Range, medians, and *p*-value for differences in WBC, Fibrinogen, DDs, DDs/WBC, PT, and PT/WBC between acute promyelocytic leukemia (APL) and “APL-like” NPM1-mutated cases.

	APL (28)	“APL-like” NPM1m (26)	
**WBC (G/L)**	2.25 (0.4–69.6)	53.25 (1.3–251.5)	*p* < 0.001
**Fibrinogen (g/L)**	1.5 (0.4–3.3)	4.5 (0.9–9.6)	*p* < 0.001
**DD (mg/L)**	14.6 (2.3–80)	9.4 (0.6–80)	*p* = 0.57
**DD/WBC**	5.44 (0.23–160)	0.43 (0.04–4.92)	*p* < 0.001
**PT (sec)**	13.1 (11.3–22.1)	11.7 (10.5–18.2)	*p* = 0.049
**PT/WBC**	4.83 (0.21–33.25)	0.23 (0.06–8.54)	*p* < 0.001

## Data Availability

Raw datasets of this work are not in a publicly archived dataset due to institutional restrictions and capacity reasons, but can be obtained after request to the corresponding author.

## Supplementary Materials

The following supporting information can be downloaded at: https://www.mdpi.com/article/10.3390/biomedicines12102282/s1, Figure S1: Typical immunophenotype of an “acute-promyelocytic leukemia-like” NPM1-mutated AML case of our cohort. Negativity for CD34 and HLA-DR (“double-negative” immunophenotype, similarly to APL). Negativity for the monoblastic/monocytic marker CD64. Clear and typical positivity for CD33; Figure S2: Mutation cascade. Each column represents one NPM1-mutated AML case. All NPM1-mutated AML cases are included (“APL-like”, “non-APL-like”). A red box means presence of mutation of the respective gene, an empty box absence of mutation, a grey box gene not included in the panel. Number noted in some of the red box means more than one mutation in the respective gene.
