# Supplementary material for: Characteristics and Prognosis of “Acute Promyelocytic Leukemia-like” Nucleophosmin-1-Mutated Acute Myeloid Leukemia in a Retrospective Patient Cohort
Source: Biomedicines. 2024 Oct 9;12(10):2282. doi: 10.3390/biomedicines12102282 (PMC11505509; doi:10.3390/biomedicines12102282)
Supplement: Supplementary file 1 [file biomedicines-12-02282-s001.zip › biomedicines-3239283-supplementary.pdf]

Figure S1.

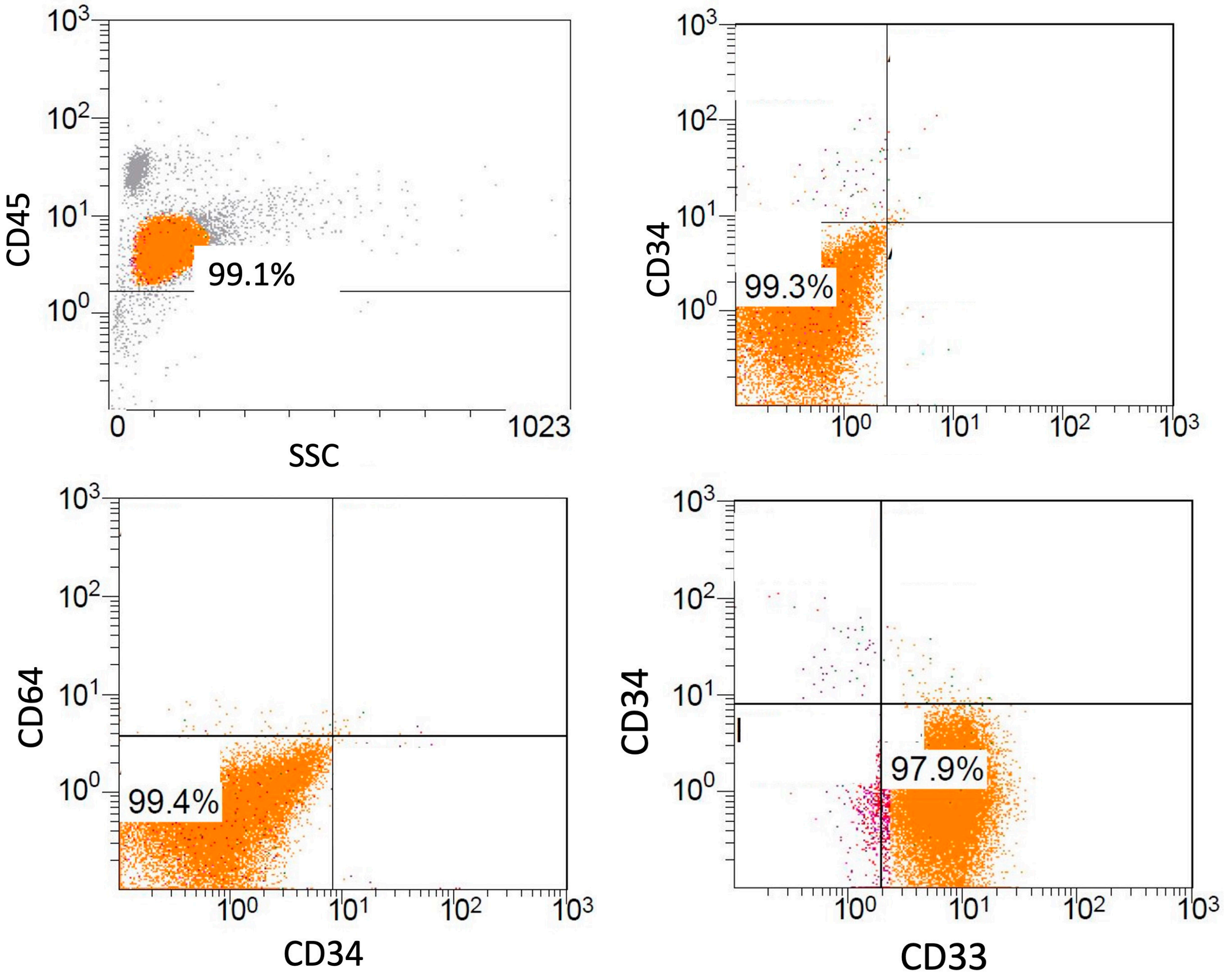

Typical immunophenotype of an "acute-promyelocytic leukemia-like" NPM1-mutated AML case of our cohort. Negativity for CD34 and HLA-DR ("double-negative" immunophenotype, similarly to APL). Negativity for the monoblastic/monocytic marker CD64. Clear and typical positivity for CD33.

Figure S2.

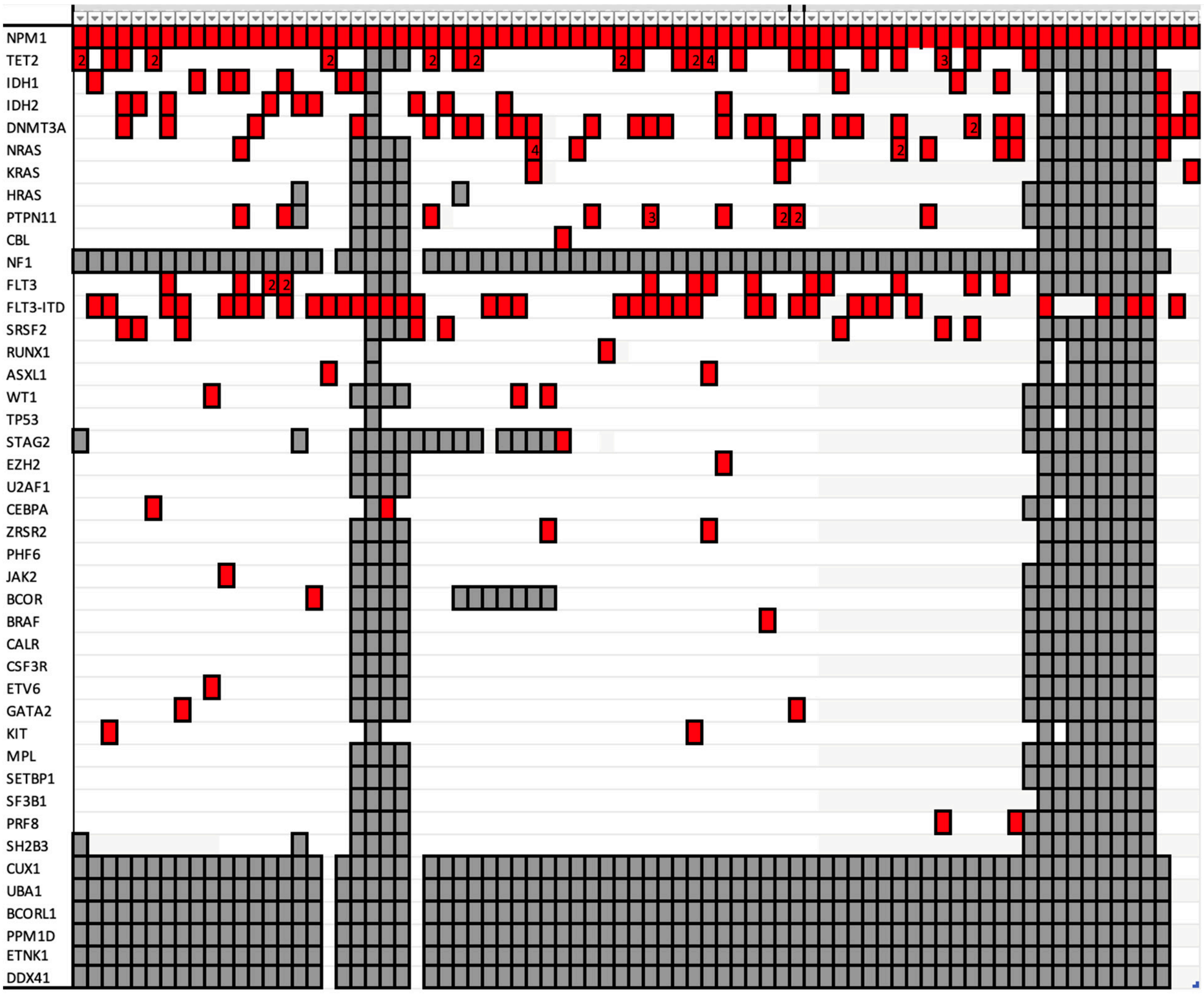

Mutation cascade. Each column represents one NPM1-mutated AML case. All NPM1-mutated AML cases are included (“APL-like”, “non-APL-like”). A red box means presence of mutation of the respective gene, an empty box absence of mutation, a grey box gene not included in the panel. Number noted in some of the red box means more than one mutation in the respective gene.
